# [2,6-Bis(dimethyl­amino­meth­yl)phen­yl]selenium bromide monohydrate

**DOI:** 10.1107/S1600536810008019

**Published:** 2010-03-06

**Authors:** Richard A. Varga, Monica Kulcsar, Anca Silvestru

**Affiliations:** a Faculty of Chemistry and Chemical Engineering, Babes-Bolyai University, Arany Janos Str. no. 11, RO-400028, Cluj Napoca, Romania; b Departamento de Química Inorgánica, Instituto de Ciencia de, Materiales de Aragón, Universidad de Zaragoza–CSIC, E-50009 Zaragoza, Spain

## Abstract

In the title hydrated molecular salt, C_12_H_19_N_2_Se^+^·Br^−^·H_2_O, the two independent bromide anions lie on a twofold rotation axis. Strong intra­molecular N→Se inter­actions [2.185 (3) and 2.181 (3) Å] are established by both N atoms of the organic group in the cation, in *trans* positions to each other, with an N—Se—N angle of 161.6 (1)°, resulting in a T-shaped (*C*,*N*,*N*′)Se core. In the crystal, dimeric associations are formed by Br⋯Se [3.662 (2) Å] and Br⋯H inter­actions [2.56 (6) and 2.63 (7) Å] involving two bromide anions, two cations and two water mol­ecules.

## Related literature

For related selenium and tellurium compounds, see: Drake *et al.* (2001*a* ▶,*b* ▶); Deleanu *et al.* (2002 ▶); Kulcsar *et al.* (2005 ▶, 2007 ▶); Beleaga *et al.* (2009 ▶); Fujihara *et al.* (1995 ▶). For van der Waals radii, see: Emsley (1994 ▶). 
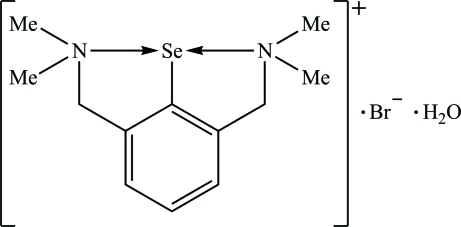


## Experimental

### 

#### Crystal data

C_12_H_19_N_2_Se^+^·Br^−^·H_2_O
*M* *_r_* = 368.18
Monoclinic, 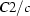

*a* = 15.1494 (14) Å
*b* = 11.3182 (10) Å
*c* = 18.8083 (17) Å
β = 110.475 (2)°
*V* = 3021.2 (5) Å^3^
*Z* = 8
Mo *K*α radiation
μ = 5.12 mm^−1^
*T* = 297 K
0.31 × 0.29 × 0.09 mm

#### Data collection

Bruker SMART APEX CCD area-detector diffractometer
Absorption correction: multi-scan (*SADABS*; Bruker, 2000 ▶) *T*_min_ = 0.300, *T*_max_ = 0.656
11856 measured reflections
3093 independent reflections
2696 reflections with *I* > 2/s(*I*)
*R*_int_ = 0.050

#### Refinement

*R*[*F* ^2^ > 2σ(*F* ^2^)] = 0.039
*wR*(*F* ^2^) = 0.086
*S* = 1.09
3093 reflections
167 parameters
H atoms treated by a mixture of independent and constrained refinement
Δρ_max_ = 0.49 e Å^−3^
Δρ_min_ = −0.53 e Å^−3^

### 

Data collection: *SMART* (Bruker, 2000 ▶); cell refinement: *SAINT-Plus* (Bruker, 2001 ▶); data reduction: *SAINT-Plus*; program(s) used to solve structure: *SHELXS97* (Sheldrick, 2008 ▶); program(s) used to refine structure: *SHELXL97* (Sheldrick, 2008 ▶); molecular graphics: *ORTEP-3* (Farrugia, 1997 ▶) and *DIAMOND* 3 (Brandenburg & Putz, 2006 ▶); software used to prepare material for publication: *publCIF* (Westrip, 2010 ▶).

## Supplementary Material

Crystal structure: contains datablocks I, global. DOI: 10.1107/S1600536810008019/dn2538sup1.cif

Structure factors: contains datablocks I. DOI: 10.1107/S1600536810008019/dn2538Isup2.hkl

Additional supplementary materials:  crystallographic information; 3D view; checkCIF report

## Figures and Tables

**Table 1:** Hydrogen-bond geometry (Å, °)

*D*—H⋯*A*	*D*—H	H⋯*A*	*D*⋯*A*	*D*—H⋯*A*
O1—H1⋯Br2	0.78 (6)	2.56 (6)	3.340 (5)	175 (5)
O1—H2⋯Br1	0.78 (6)	2.63 (7)	3.406 (5)	176 (7)

**Fig. 1.**
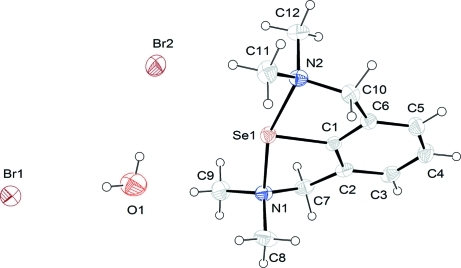
: View of title compound showing the atom-numbering scheme at 30% probability thermal ellipsoids.

**Fig. 2.**
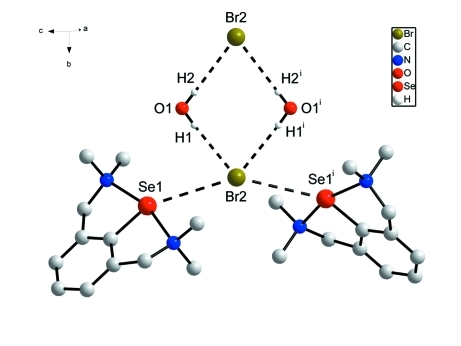
: Intra- and intermolecular interactions in the title compound (dashed lines; only H atoms involved in interactions are shown) [symmetry code: (i) = 1-x, y, 1.5-z].

## supplementary crystallographic information

### Comment

In the last years our interest was focused on the synthesis, structural characterization and chemical reactivity of some new hypervalent organoselenium and organotellurium derivatives containing aryl groups with pendant arms, *e.g. *2-(Me_2_NCH_2_)C_6_H_4_, 2-[O(CH_2_CH_2_)_2_NCH_2_]C_6_H_4_ and 2-[MeN(CH_2_CH_2_)_2_NCH_2_]C_6_H_4_ (Drake *et al.*, 2001a,b, Deleanu *et al.*,2002, Kulcsar *et al.*, 2005, 2007, Beleaga *et al.*, 2009).

The crystal of the title compound contains a mixture of [{2,6-(Me_2_NCH_2_)_2_C_6_H_3_}Se]^+^ cations and [Br]^-^ anions and crystallizes with a water molecule. The two independent bromine anions lie on a two-fold rotation axis. Both pendant arms of the organic group attached to selenium establish strong intramolecular N→Se interactions [Se—N1 = 2.185 (3) Å, Se1—N2 = 2.181 (3) Å], *trans* one to the other [N1—Se1—N2 = 161.6 (1)°], thus resulting in the increase of the coordination number at Se to three (Fig. 1). The Se—N distances are of the same magnitude as found in the cation of [{2,6-(Me_2_NCH_2_)_2_C_6_H_3_}Se]^+^[PF_6_]^-^ [Se1—N1 = 2.180 Å; Se1—N2 = 2.154 Å] (Fujihara *et al.*, 1995). This results in a distorted T-shaped (*C,N,N'*)Se core [C1—Se1—N1 = 80.5 (1)°, C1—Se1—N2 = 81.2 (1)°], the distortion being mainly due to the constraints imposed by the two SeC_3_N five-membered chelate rings.

An unusual dimer association is formed between two cations, two bromine atoms and two water molecules (Fig. 2). The Br2 atom is involved in bridging two selenium atoms and the interatomic Se1—Br2 distances [3.662 (2) Å] are much longer than the sum of the corresponding covalent radii [Σ*r*_cov_(Se,Br) ca. 2.31 Å], but shorter than the sum of the van der Waals radii [Σ*r*_vdW_(Se,Br) ca. 3.95 Å] (Emsley, 1994), consistent with an electrostatic anion-cation interaction. This interaction is directed *trans *to the selenium-carbon bond in the cation [C1—Se1—Br2 = 154.4 (1)°], thus resulting in a distorted square-planar environment around the chalcogen atom. The water molecules bridge the Br1 and Br2 anions through Br···H hydrogen bonding [Br1···H1 = 2.56 (6) Å, Br2···H2 = 2.63 (7) Å].

### Experimental

[2,6-(Me_2_NCH_2_)_2_C_6_H_3_]SeBr was obtained by oxidizing [2,6-(Me_2_NCH_2_)_2_C_6_H_3_]_2_Se_2 _with elemental bromine.

The attempt to grow crystals of [2,6-(Me_2_NCH_2_)_2_C_6_H_3_]SeBr from a methylene dichloride / n-hexane mixture (1:5, v/v) in open atmosphere led to the isolation of [2,6-(Me_2_NCH_2_)_2_C_6_H_3_]SeBr.H_2_O.

### Refinement

All hydrogen atoms were placed in calculated positions using a riding model, with C—H = 0.93-0.97 Å and with U_iso_= 1.5U_eq_ (C) for methyl H and U_iso_= 1.2U_eq_ (C) for aryl H. The methyl groups were allowed to rotate but not to tip. Hydrogen atoms from the water molecule were found from difference map and refined to O—H distances of 0.78 (6) Å.

### Crystal data

**Table:**

C_12_H_19_N_2_Se^+^·Br^−^·H_2_O	*F*(000) = 1472
*M**_r_* = 368.18	*D*_x_ = 1.619 Mg m^−^^3^
Monoclinic, *C*2/*c*	Mo *K*α radiation, λ = 0.71073 Å
Hall symbol: -C 2yc	Cell parameters from 3226 reflections
*a* = 15.1494 (14) Å	θ = 2.3–25.2°
*b* = 11.3182 (10) Å	µ = 5.12 mm^−^^1^
*c* = 18.8083 (17) Å	*T* = 297 K
β = 110.475 (2)°	Block, colourless
*V* = 3021.2 (5) Å^3^	0.31 × 0.29 × 0.09 mm
*Z* = 8	

### Data collection

**Table:**

Bruker SMART APEX CCD area-detector diffractometer	3093 independent reflections
Radiation source: fine-focus sealed tube	2696 reflections with *I* > 2/s(*I*)
graphite	*R*_int_ = 0.050
phi and ω scans	θ_max_ = 26.4°, θ_min_ = 2.3°
Absorption correction: multi-scan (*SADABS*; Bruker, 2000)	*h* = −18→18
*T*_min_ = 0.300, *T*_max_ = 0.656	*k* = −14→14
11856 measured reflections	*l* = −23→23

### Refinement

**Table:**

Refinement on *F*^2^	Primary atom site location: structure-invariant direct methods
Least-squares matrix: full	Secondary atom site location: difference Fourier map
*R*[*F*^2^ > 2σ(*F*^2^)] = 0.039	Hydrogen site location: inferred from neighbouring sites
*wR*(*F*^2^) = 0.086	H atoms treated by a mixture of independent and constrained refinement
*S* = 1.09	*w* = 1/[σ^2^(*F*_o_^2^) + (0.0287*P*)^2^ + 3.8997*P*] where *P* = (*F*_o_^2^ + 2*F*_c_^2^)/3
3093 reflections	(Δ/σ)_max_ = 0.001
167 parameters	Δρ_max_ = 0.49 e Å^−^^3^
0 restraints	Δρ_min_ = −0.53 e Å^−^^3^

### Special details

**Table:**

Geometry. All esds (except the esd in the dihedral angle between two l.s. planes) are estimated using the full covariance matrix. The cell esds are taken into account individually in the estimation of esds in distances, angles and torsion angles; correlations between esds in cell parameters are only used when they are defined by crystal symmetry. An approximate (isotropic) treatment of cell esds is used for estimating esds involving l.s. planes.
Refinement. Refinement of *F*^2^ against ALL reflections. The weighted *R*-factor wR and goodness of fit *S* are based on *F*^2^, conventional *R*-factors *R* are based on *F*, with *F* set to zero for negative *F*^2^. The threshold expression of *F*^2^ > σ(*F*^2^) is used only for calculating *R*-factors(gt) etc. and is not relevant to the choice of reflections for refinement. *R*-factors based on *F*^2^ are statistically about twice as large as those based on *F*, and *R*- factors based on ALL data will be even larger.

### Fractional atomic coordinates and isotropic or equivalent isotropic displacement parameters (Å^2^)

**Table:**

	*x*	*y*	*z*	*U*_iso_*/*U*_eq_	
Br1	0.5000	0.26510 (5)	0.7500	0.05461 (17)	
Br2	0.5000	0.73811 (6)	0.7500	0.0747 (2)	
C1	0.3337 (2)	0.9300 (3)	0.93573 (18)	0.0319 (7)	
C2	0.3774 (2)	0.9329 (3)	1.01348 (18)	0.0372 (7)	
C6	0.2603 (2)	1.0046 (3)	0.89677 (19)	0.0346 (7)	
C10	0.2169 (2)	0.9828 (3)	0.81308 (19)	0.0408 (8)	
H10A	0.1640	0.9290	0.8028	0.049*	
H10B	0.1939	1.0565	0.7868	0.049*	
C7	0.4589 (3)	0.8503 (3)	1.04538 (19)	0.0444 (8)	
H7A	0.5173	0.8906	1.0505	0.053*	
H7B	0.4622	0.8232	1.0952	0.053*	
C8	0.3799 (3)	0.6604 (3)	1.0050 (3)	0.0572 (11)	
H8A	0.4077	0.6257	1.0544	0.086*	
H8B	0.3677	0.5999	0.9670	0.086*	
H8C	0.3218	0.6984	1.0014	0.086*	
C9	0.5358 (3)	0.6906 (4)	0.9999 (2)	0.0561 (10)	
H9A	0.5780	0.7482	0.9919	0.084*	
H9B	0.5246	0.6294	0.9624	0.084*	
H9C	0.5635	0.6570	1.0496	0.084*	
C12	0.3495 (3)	1.0235 (3)	0.7709 (2)	0.0495 (9)	
H12A	0.4006	0.9872	0.7599	0.074*	
H12B	0.3744	1.0733	0.8147	0.074*	
H12C	0.3126	1.0702	0.7282	0.074*	
C11	0.2469 (3)	0.8570 (4)	0.7181 (2)	0.0525 (9)	
H11A	0.2098	0.9056	0.6767	0.079*	
H11B	0.2073	0.7982	0.7286	0.079*	
H11C	0.2957	0.8189	0.7050	0.079*	
C5	0.2301 (3)	1.0860 (3)	0.9381 (2)	0.0454 (9)	
H5	0.1810	1.1374	0.9134	0.054*	
C4	0.2730 (3)	1.0911 (3)	1.0160 (2)	0.0505 (9)	
H4	0.2525	1.1462	1.0434	0.061*	
C3	0.3460 (3)	1.0155 (3)	1.0540 (2)	0.0473 (9)	
H3	0.3740	1.0198	1.1065	0.057*	
N1	0.4453 (2)	0.7484 (2)	0.99307 (16)	0.0385 (7)	
N2	0.28962 (19)	0.9310 (2)	0.78604 (15)	0.0372 (6)	
O1	0.4714 (3)	0.5053 (4)	0.8489 (2)	0.0793 (11)	
Se1	0.37778 (2)	0.82185 (3)	0.879443 (18)	0.03542 (12)	
H1	0.480 (4)	0.562 (5)	0.828 (3)	0.09 (2)*	
H2	0.475 (5)	0.451 (6)	0.825 (4)	0.12 (3)*	

### Atomic displacement parameters (Å^2^)

**Table:**

	*U*^11^	*U*^22^	*U*^33^	*U*^12^	*U*^13^	*U*^23^
Br1	0.0623 (4)	0.0495 (3)	0.0552 (4)	0.000	0.0246 (3)	0.000
Br2	0.0914 (5)	0.0596 (4)	0.0986 (6)	0.000	0.0651 (5)	0.000
C1	0.0345 (17)	0.0321 (16)	0.0323 (17)	−0.0077 (13)	0.0157 (14)	−0.0033 (13)
C2	0.0410 (19)	0.0415 (18)	0.0301 (18)	−0.0085 (15)	0.0139 (15)	0.0012 (14)
C6	0.0337 (17)	0.0329 (16)	0.0367 (18)	−0.0046 (13)	0.0117 (14)	−0.0018 (13)
C10	0.0355 (18)	0.0434 (19)	0.0379 (19)	0.0018 (15)	0.0057 (15)	0.0008 (15)
C7	0.043 (2)	0.057 (2)	0.0302 (18)	−0.0040 (17)	0.0092 (15)	0.0064 (16)
C8	0.045 (2)	0.050 (2)	0.073 (3)	−0.0058 (17)	0.016 (2)	0.021 (2)
C9	0.040 (2)	0.065 (3)	0.061 (3)	0.0149 (19)	0.0143 (19)	0.014 (2)
C12	0.056 (2)	0.053 (2)	0.040 (2)	−0.0079 (18)	0.0165 (18)	0.0080 (17)
C11	0.058 (2)	0.055 (2)	0.036 (2)	−0.0003 (19)	0.0076 (18)	−0.0115 (17)
C5	0.047 (2)	0.0364 (18)	0.056 (2)	0.0009 (16)	0.0221 (18)	−0.0025 (16)
C4	0.063 (2)	0.043 (2)	0.055 (2)	−0.0016 (19)	0.033 (2)	−0.0114 (18)
C3	0.060 (2)	0.054 (2)	0.0317 (19)	−0.0089 (19)	0.0210 (18)	−0.0050 (16)
N1	0.0319 (15)	0.0426 (16)	0.0395 (16)	0.0017 (12)	0.0108 (13)	0.0081 (12)
N2	0.0412 (16)	0.0396 (15)	0.0273 (14)	−0.0040 (12)	0.0076 (12)	−0.0005 (12)
O1	0.106 (3)	0.077 (3)	0.046 (2)	−0.007 (2)	0.0145 (19)	0.0004 (19)
Se1	0.03654 (19)	0.03788 (19)	0.03126 (19)	0.00273 (14)	0.01113 (14)	−0.00128 (14)

### Geometric parameters (Å, °)

**Table:**

C1—C2	1.379 (4)	C9—H9B	0.9600
C1—C6	1.384 (5)	C9—H9C	0.9600
C1—Se1	1.887 (3)	C12—N2	1.478 (4)
C2—C3	1.391 (5)	C12—H12A	0.9600
C2—C7	1.497 (5)	C12—H12B	0.9600
C6—C5	1.382 (5)	C12—H12C	0.9600
C6—C10	1.499 (5)	C11—N2	1.474 (4)
C10—N2	1.487 (4)	C11—H11A	0.9600
C10—H10A	0.9700	C11—H11B	0.9600
C10—H10B	0.9700	C11—H11C	0.9600
C7—N1	1.483 (5)	C5—C4	1.381 (5)
C7—H7A	0.9700	C5—H5	0.9300
C7—H7B	0.9700	C4—C3	1.383 (5)
C8—N1	1.476 (4)	C4—H4	0.9300
C8—H8A	0.9600	C3—H3	0.9300
C8—H8B	0.9600	N1—Se1	2.185 (3)
C8—H8C	0.9600	N2—Se1	2.180 (3)
C9—N1	1.484 (5)	O1—H1	0.78 (6)
C9—H9A	0.9600	O1—H2	0.78 (6)
			
C2—C1—C6	123.1 (3)	H12A—C12—H12B	109.5
C2—C1—Se1	118.5 (2)	N2—C12—H12C	109.5
C6—C1—Se1	118.4 (2)	H12A—C12—H12C	109.5
C1—C2—C3	117.9 (3)	H12B—C12—H12C	109.5
C1—C2—C7	115.7 (3)	N2—C11—H11A	109.5
C3—C2—C7	126.3 (3)	N2—C11—H11B	109.5
C5—C6—C1	118.1 (3)	H11A—C11—H11B	109.5
C5—C6—C10	126.1 (3)	N2—C11—H11C	109.5
C1—C6—C10	115.6 (3)	H11A—C11—H11C	109.5
N2—C10—C6	108.6 (3)	H11B—C11—H11C	109.5
N2—C10—H10A	110.0	C4—C5—C6	119.9 (3)
C6—C10—H10A	110.0	C4—C5—H5	120.1
N2—C10—H10B	110.0	C6—C5—H5	120.1
C6—C10—H10B	110.0	C5—C4—C3	121.2 (3)
H10A—C10—H10B	108.4	C5—C4—H4	119.4
N1—C7—C2	108.4 (3)	C3—C4—H4	119.4
N1—C7—H7A	110.0	C4—C3—C2	119.8 (3)
C2—C7—H7A	110.0	C4—C3—H3	120.1
N1—C7—H7B	110.0	C2—C3—H3	120.1
C2—C7—H7B	110.0	C8—N1—C7	111.6 (3)
H7A—C7—H7B	108.4	C8—N1—C9	109.8 (3)
N1—C8—H8A	109.5	C7—N1—C9	112.1 (3)
N1—C8—H8B	109.5	C8—N1—Se1	107.2 (2)
H8A—C8—H8B	109.5	C7—N1—Se1	105.19 (19)
N1—C8—H8C	109.5	C9—N1—Se1	110.7 (2)
H8A—C8—H8C	109.5	C11—N2—C12	110.4 (3)
H8B—C8—H8C	109.5	C11—N2—C10	111.5 (3)
N1—C9—H9A	109.5	C12—N2—C10	111.4 (3)
N1—C9—H9B	109.5	C11—N2—Se1	109.6 (2)
H9A—C9—H9B	109.5	C12—N2—Se1	108.2 (2)
N1—C9—H9C	109.5	C10—N2—Se1	105.46 (19)
H9A—C9—H9C	109.5	H1—O1—H2	107 (6)
H9B—C9—H9C	109.5	C1—Se1—N2	81.21 (12)
N2—C12—H12A	109.5	C1—Se1—N1	80.47 (12)
N2—C12—H12B	109.5	N2—Se1—N1	161.56 (11)
			
C6—C1—C2—C3	−0.4 (5)	C2—C7—N1—Se1	34.9 (3)
Se1—C1—C2—C3	178.1 (2)	C6—C10—N2—C11	152.9 (3)
C6—C1—C2—C7	−176.8 (3)	C6—C10—N2—C12	−83.3 (3)
Se1—C1—C2—C7	1.8 (4)	C6—C10—N2—Se1	33.9 (3)
C2—C1—C6—C5	0.4 (5)	C2—C1—Se1—N2	−166.9 (3)
Se1—C1—C6—C5	−178.1 (2)	C6—C1—Se1—N2	11.7 (2)
C2—C1—C6—C10	−175.7 (3)	C2—C1—Se1—N1	15.2 (2)
Se1—C1—C6—C10	5.7 (4)	C6—C1—Se1—N1	−166.2 (3)
C5—C6—C10—N2	155.8 (3)	C11—N2—Se1—C1	−146.0 (3)
C1—C6—C10—N2	−28.4 (4)	C12—N2—Se1—C1	93.5 (2)
C1—C2—C7—N1	−26.9 (4)	C10—N2—Se1—C1	−25.8 (2)
C3—C2—C7—N1	157.1 (3)	C11—N2—Se1—N1	−139.3 (3)
C1—C6—C5—C4	−0.1 (5)	C12—N2—Se1—N1	100.2 (4)
C10—C6—C5—C4	175.6 (3)	C10—N2—Se1—N1	−19.1 (4)
C6—C5—C4—C3	−0.2 (6)	C8—N1—Se1—C1	90.8 (2)
C5—C4—C3—C2	0.2 (6)	C7—N1—Se1—C1	−28.1 (2)
C1—C2—C3—C4	0.1 (5)	C9—N1—Se1—C1	−149.4 (3)
C7—C2—C3—C4	176.0 (3)	C8—N1—Se1—N2	84.2 (4)
C2—C7—N1—C8	−81.0 (3)	C7—N1—Se1—N2	−34.8 (4)
C2—C7—N1—C9	155.3 (3)	C9—N1—Se1—N2	−156.1 (3)

### Hydrogen-bond geometry (Å, °)

**Table:**

*D*—H···*A*	*D*—H	H···*A*	*D*···*A*	*D*—H···*A*
O1—H1···Br2	0.78 (6)	2.56 (6)	3.340 (5)	175 (5)
O1—H2···Br1	0.78 (6)	2.63 (7)	3.406 (5)	176 (7)
